# Supplementary material for: An Atomistic Insight into Moiré Reconstruction in Twisted Bilayer Graphene beyond the Magic Angle
Source: ACS Appl Eng Mater. 2023 Mar 13;1(3):970–82. doi: 10.1021/acsaenm.2c00259 (PMC10043875; doi:10.1021/acsaenm.2c00259)
Supplement: Supplementary file 1 — em2c00259_si_001.pdf [file em2c00259_si_001.pdf]

Supporting Information

An atomistic insight to moiré reconstruction in Twisted Bilayer Graphene beyond magic angle

Aditya Dey,[1,a)] Shoieb Ahmed Chowdhury,[1] Tara Peña,[2] Sobhit Singh,[1] Stephen M. Wu,[2,b)] and Hesam Askari[3]

[1)] *Department of Mechanical Engineering, University of Rochester, Rochester, New York*

[2)] *Department of Electrical and Computer Engineering, University of Rochester, Rochester, New York*

[3)] *Department of Mechanical Engineering, University of Rochester, New York*

[a)] Electronic mail: adey2@ur.rochester.edu

[b)] Department of Physics and Astronomy, University of Rochester, Rochester, New York

I. COMPUTATIONAL AND THEORETICAL METHODS

A. DFT calculations

The real space lattices of TBG systems were constructed using ATOMISTIX TOOLKIT (QuantumATK) commercial package. All the first principles simulations were conducted with generalized gradient approximation (GGA)[1,2] assimilated in Quantum Espresso open source package. The Perdew-Burke-Ernzerhof (PBE) form and GGA have been used as the exchange-correlation functional[3]. Ultrasoft pseudopotentials have described ion-electron interactions for carbon atoms in TBGs. The vdW interaction has also been incorporated using the semi-empirical Grimme functional[4]. Wavefunctions are expanded using a plane wave basis set with an energy cutoff and charge density of 55 Ry and 450 Ry, respectively. We used $14 \times 14 \times 1$ k-point grid within Monkhorst-Pack[5,6] scheme to sample the reciprocal space Brillouin zone. The structures were optimized until all the atomic forces were less than 0.01 eV/Å. The in-plane lattice constants were relaxed, including the non-periodic out-of-plane lattice (25 Å space) to elude interactions in that direction. Phonon dispersion spectra of all TBG structures were simulated using self-consistent density functional perturbation theory (DFPT)[7,8]. The dynamical matrices were first computed on an adequate q-point grid. The inter-atomic constants used in computing the phonon dispersion were obtained from the Fourier interpolation of these dynamical matrices.

B. MS simulations

Molecular statics simulations were done using LAMMPS open-source software. The unstrained, DFT-relaxed TBG moiré lattice was transformed into an orthogonal cell with approximate dimensions of 32 nm $\times$ 20 nm for all the TBG structures. The number of MPs generated in each structure is dependent on the twist angle; for example $\theta = 6°$has 72 MPs, and $\theta = 13.2°$has 288 MPs, respectively. A vacuum space of 50 Å is inserted along the out-plane direction to avoid interactions with the periodic images. Hydrogen passivation was done along the free surfaces to obtain the most stable structure. The TBG structures were minimized using a conjugate gradient energy minimization method to have minimum energy configurations. A reactive empirical bond order (REBO) potential was used for the intralayer covalent bonds[9]. For the interlayer van der Waals interaction, a registry-dependent

Kolmogorov-Crespi (KC) potential[10] was selected. As TBG contains different local stacking configurations, an interatomic potential that considers registry different than equilibrium minimum energy stacking is needed[11,12]. Subsequently, we loaded the structure with constant incremental strain to the top layer. We limit the magnitude of applied strain to 1% for impeding our analysis within the contended boundaries of the experimental capability of straining such systems[13,14]. Between each loading step, the atoms of the top layer were kept stationary at the applied strain level, and energy minimization was performed. The snapshots of the structure at different strain magnitudes were taken in Ovito open visualization tool[15].

C. BOLS formulation

The BOLS notion explains the bond contraction and bond strengthening phenomena using the following expressions[16]:

$$\frac{d_z}{d_b} = C_z = \frac{2}{1 + exp[\frac{12-z}{8z}]} \tag{1}$$

$$E_z = \frac{E_b}{C_z^m} \tag{2}$$

Here, the subscripts z and b respectively represent the coordination number (CN) of a particular atomic structure and its bulk counterpart as a standard. The terms d and E denote bond length and bond energy, respectively. C_z represents the bond contraction coefficient that varies with atomic structures having different z. The bond nature index is denoted by m which is 2.56 for carbon bonds[17]. Since we are dealing with graphitic structures in this study, we consider the bulk counterpart as diamond. Using the bond length of the diamond ($d_b = 1.54$Å) and bond lengths d_z for each stacking configuration, we can calculate C_z and z for each configuration using equations (1) and (2). Again using the relation given in equation (2), we can calculate the bond energy for each stacking. For diamond, the single C-C bond energy can be obtained from its total cohesive energy, known to us, i.e., $E_b = 0.614$ eV[17]. Having known z, d_z, and E_z, we calculate the β pre-factor values for each stacking using equation (2). The relation stated in equation 1 in the main text can be derived by equating the vibrational energy of a harmonic system to the first-order approximated Taylor series of its interatomic potential as[16]:

$$\frac{1}{2}\mu(\Delta\omega)^2x^2 \cong \frac{1}{2}\frac{\delta u(r)}{\delta r^2}x^2 \propto \frac{1}{2}\frac{E_z}{{d_z}^2}x^2 \tag{3}$$

$$\Rightarrow \Delta\omega \propto \frac{z}{d_z}\sqrt{\frac{E_z}{\mu}}$$

The BOLS correlation is also used to estimate the phonon frequencies pertaining to Raman G-band peaks. To achieve this, we perform some steps of mathematical interpolation for equation (3). We can write the equation as $\Delta\omega^G = \omega^G_{TBG} - \omega^G_{ref} = k\,(\beta)$, where ω^G_{ref} is the G band frequency of any reference material. Now we can calculate ω^G_{ref} for each TBG system with respect to their bulk counterpart (diamond) by comparing respective β pre-factors as, $\frac{\omega^G_{TBG} - \omega^G_{ref}}{\omega_{diamond} - \omega^G_{ref}} = \frac{\beta_{TBG}}{\beta_{diamond}}$. After obtaining ω^G_{ref}, we can exercise $\omega^{G,\epsilon=0}_{TBG}$ (ω^G_{TBG} at zero strain) and β pre-factors of strained and unstrained TBG systems to estimate their G-band frequency in strained configuration ($\omega^{G,\epsilon}_{TBG}$), as $\frac{\omega^{G,\epsilon=0}_{TBG} - \omega^G_{ref}}{\omega^{G,\epsilon}_{TBG} - \omega^G_{ref}} = \frac{\beta^{\epsilon=0}_{TBG}}{\beta^{\epsilon}_{TBG}}$. Operating this individually for top and bottom layers, we can obtain their G-peak frequencies for both directions and various magnitudes of applied strain. The β^{ϵ}_{TBG} values for the strained top layer are listed in Table SIV. Since the bottom layer remains unstrained, we observe negligible differences between their β pre-factor values for strained and unstrained configurations.

II. GEOMETRIC ANALYSIS OF STRAINED TBG

We deduce the expressions of their reciprocal lattice ($\vec{q}$) vectors to quantify the structural changes in strained MSCs[18,19]. The reciprocal lattice vectors of TBG moiré lattices[20] ($\vec{q}$) is given as $\vec{q} = \vec{b'} - \vec{b}$, where $\vec{b'}$ and $\vec{b}$ denote the reciprocal lattice vectors of the rotated top layer and bottom layer in a TBG structure respectively. The length of moiré pattern (MP), L_m can be derived using the magnitude of $\vec{q}$ vector as $L_m = \frac{4\pi}{\sqrt{3}|\vec{q}|}$. When strain is applied to the top layer, the mathematical expression of its reciprocal lattice vector[19] ($\vec{b^{\varepsilon}_i}$) can be written as $\vec{b^{\varepsilon}_i} = (\vec{I} + \vec{S})^{-1}\vec{b'_i}$, where $\vec{I}$ is the identity matrix and $\vec{S}$ denotes the strain tensor which can be written as the following for the case of uniaxial tension,

$$\vec{S} = \begin{pmatrix} \varepsilon & 0 \\ 0 & -\nu\varepsilon \end{pmatrix}$$

Here, ε is the nominal strain applied and ν denotes the Poisson's ratio. So, the reciprocal lattice vector of TBG with heterostrain can be expressed as $\vec{q^{\varepsilon}_i} = \vec{b^{\varepsilon}_i} - \vec{b_i}$. As shown in

Fig 1(e) in the main text, the boundaries of MPs resemble a hexagon and we can draw a triangle (ΔABC) with $\vec{AB}$ and $\vec{BC}$ as the MP lattice vectors and α being the angle between them ($\alpha = 60°$, $\phi = 120°$). The variation of α and ϕ with the applied strain is shown in Fig. S2. With uniaxial tension, we see a monotonic decrease in these angles and vice-versa for uniaxial compression. The changes in expressions of $\vec{q}$ vectors are associated with the geometrical changes enforced upon hetero-straining these systems.

III. EXPLANATION OF STACKING IDENTIFICATION METHOD (FOR UNSTRAINED AND STRAINED SYSTEMS)

Firstly, we identified atoms that should be classified as 'AA' type using ILS. As observed in the main text Fig. 1(c) and (d), the spacing between two layers of TBG varies due to out-of-plane displacements of atoms. The ILS of equilibrium structures follows this trend: AA > SP > AB. Hence, in a TBG system, the maximum ILS (d_{max}) corresponds to AA region, and the minimum distance (d_{min}) represents the AB region. It is observed that d_{max} and d_{min} vary with increasing twist angle up to 21°, after which we noticed a plateaued regime[21]. This results from the depletion of perfectly stacked AA and AB configurations, as the length of the MPs, reduces with increasing θ. We obtained the maximum and minimum magnitudes of d_{max} (3.589Å and 3.475Å) and d_{min} (3.456Å and 3.338Å). Using the lower bound of d_{max} for all the twist angles, i.e., 3.475Å, we classified the atoms with local ILS greater than 3.475Å as 'AA' stacking type. On the other hand, considering the upper bound of d_{min} and identifying the regions with ILS below that value as AB stacking can lead to the misclassification of AB and SP types. For the wide range of twist angles considered in this study, the ILS alone cannot provide a margin of separation for classifying AB and SP stacked atoms. To address this issue, we considered interlayer energy or ILE (per atom) in the structure. Perusing the ILE contour plot, we observed that the center of MPs has the highest energy, followed by the SP segments. The AB (or BA) has the lowest energy corresponding to the ground state configuration of BLG. But, being a per-atom quantity, the C atoms in AB stacking that are present directly on top of a C atom on the other layer show the highest ILE value, as shown in main text Fig. 2(c).

To obtain the same measure of energy for AB stacked atoms, whether they are located at the center of a lattice hexagon or at the corner, we calculated the difference of interlayer

energy of each atom with its three bonded neighbors and considered their average. The interlayer energy difference with neighboring atoms allows us to easily classify AB stacked atoms as they have the highest energy fluctuation with neighbors compared to AA or SP stacked regions where the quantity is quite uniform. To obtain a classification threshold of interlayer energy difference for AB stacking, we first calculated the soliton width of different TBG systems, i.e., the width of SP regions similarly as explained by Gargiulo et al[21]. On analyzing the path from the center of AB domain to the center of another AB (or BA) region, we traverse across the SP segment. Calculating the ILS and plotting it along the centers of triangular (AB) regions, we observed a small peak (Fig S3). This peak corresponds to the SP region and its full width at half maxima (FWHM) gives us the soliton width[21]. Considering this soliton width (varies with twist angle), we obtained the interlayer energy difference value at the boundary of SP domains. This process is repeated for different twist angles to establish a unique threshold that can be applied to any TBG system. The energy difference threshold lies in a diminutive range, 8.22-8.31 meV for the angles considered (Fig 2(e) in main text). On averaging these magnitudes, we defined a ΔE_{ILE} threshold of 8.24 meV/atom, above which an atom is classified as AB stacking type. The contour plot of TBG ($\theta = 6°$) system in main Fig 2(f) shows the outcome of applying the method where each atom has been classified as belonging to either AA or AB or SP stacked. We utilized the same approach for classifying the local domains in strained systems. Since the ILS parameter defines the out-of-plane distancing of pristine structures, it is not affected by an in-plane applied strain. However, the interlayer energy of the structure is expected to change because an externally applied strain disturbs the interlayer interactions. But since the mechanical deformation is applied globally, the local regions will experience a similar change in ILE with respect to their nearest neighbors. Hence, ΔE_{ILE} remains approximately unchanged (see Table S1).

IV. STACKING IDENTIFICATION OF RIGID STRUCTURES

We followed the same approach for reconstructed or relaxed systems to classify local regions in rigid structures. The atomistic structure of rigid TBGs (R-TBGs) differs from reconstructed systems. Since they are created by simply employing a rigid twist to a Bernal stacked bilayer graphene, they do not have a variation of interlayer spacing, which is present

in reconstructed TBGs pertaining to the formation of local stackings in the structure. When an R-TBG is modeled from Bernal stacked (or AB) graphene, it has an ILS equal to that of AB-stacked graphene throughout its structure. Hence to account for this we defined their uniform ILS, which is different from their initial geometry. We first considered their relaxed structure and obtained an average ILS value considering all the interlayer distances throughout the structure. Then, we re-modeled the rigid TBG structure by adjusting the layers with respect to the average ILS value. Since different structures have varying fractions of local interlayer regions, this average ILS changes for systems with certain twist angles. It must be noted that we have not utilized this average ILS to define any threshold to classify local atoms, rather it is used only to define the respective rigid structures. Further, following the same method as relaxed systems, we obtained their interlayer energy followed by calculating the ILE difference (ΔE_{ILE}) per atom. Now to classify the individual stackings, we referred back to the ILS and ΔE_{ILE} thresholds obtained for relaxed systems. Having known the ILS threshold for AA region (3.475 Å), we then identified the ΔE_{ILE} value at the location corresponding to that ILS value by traversing along path PQ (Fig. 2(a) main text). Then, we employed this value in ΔE_{ILE} calculation for R-TBG and specified atoms above that threshold (6.88 meV/atom) as AA. For identifying AB type, we have considered the ΔE_{ILE} threshold (8.24 meV/atom) corresponding to its location on the path PQ. Similarly, we then used that location to detect ΔE_{ILE} threshold for AB type in R-TBG structure (5.92 meV/atom, so it lies between 5.92 and 6.88 meV/atom). After classifying AB and AA, we have assigned the remaining atoms as SP. Further, we have used this same method to identify the local stackings in rigid structures of strained configurations. To model rigid systems of strained TBGs in a way that physically makes sense, we first considered the relaxed or reconstructed structure of pristine TBG. Now the top layer is stretched such that an unrelaxed hetero-strained TBG system is generated, referred to as the rigid structure in the presence of strain. Relaxing this strained structure results in a fully optimized system pertaining to the reconstructed TBG configuration with strain.

V. PHONON DISPERSION SPECTRA OF TBG AND ITS LOCAL DOMAINS

The simulations for phonon dispersion spectra were performed for $\theta = 6°$ and $13.2°$ systems. Due to the computational cost of DFT-based phonon simulations for large MPs, we computed phonon spectra only for $\theta > 4.41°$ systems. We discussed an approach using BOLS correlation to predict the Raman peaks pertaining to optical phonon modes for larger TBG systems. As described by Cocemasov et al, TBGs contain hybrid folded phonon branches that must be unfolded onto the single layer first BZ[22]. Using the PhononUnfolding package[23], we simplified the phonon spectra of TBGs along Γ-K-M-Γ high symmetry path (Fig S7 shows unfolded spectra of $\theta = 6°$). To obtain the phonon spectra of local sub-domains, we first identified the atomic positions of each local stacking as defined by our identification method and extracted the data from the main structure. Then, we calculated the average bond length l_{avg} of each configuration and deduced their respective lattice constant as $a_{stacking} = \sqrt{3}l_{avg}$. With the calculated unit cell parameters, we have computed their phonon spectrum.

VI. PHONON BAND SPLITTING WITH HETEROSTRAIN

A combination of Molecular statics and first principles simulations has been used to compute phonon dispersion spectra of TBGs with heterostrain. By freezing the obtained configuration from LAMMPS, we have extracted the atomic data of strained periodic moiré lattice and further minimized the supercell in DFT to obtain first-principles-level fidelity, followed by phonon spectra calculations. We observed strain-induced phonon band splitting due to inequivalent strain in both layers. With tension, the atomic bonds in a crystal are stretched relative to their unstrained condition. When the bond length is increased, and the force constant remains unchanged, as a result, the vibrational frequency decreases. Conversely, for compression, the bond length reduces, which leads to an increase in vibrational frequency. That is why we observe redshift and blueshift in phonon frequencies for tensile and compressive strain, respectively[24]. The redshift and blueshift of Raman G-band for $\theta = 1.08°$, shown in Fig. S9, is a good demonstration of this phenomenon.

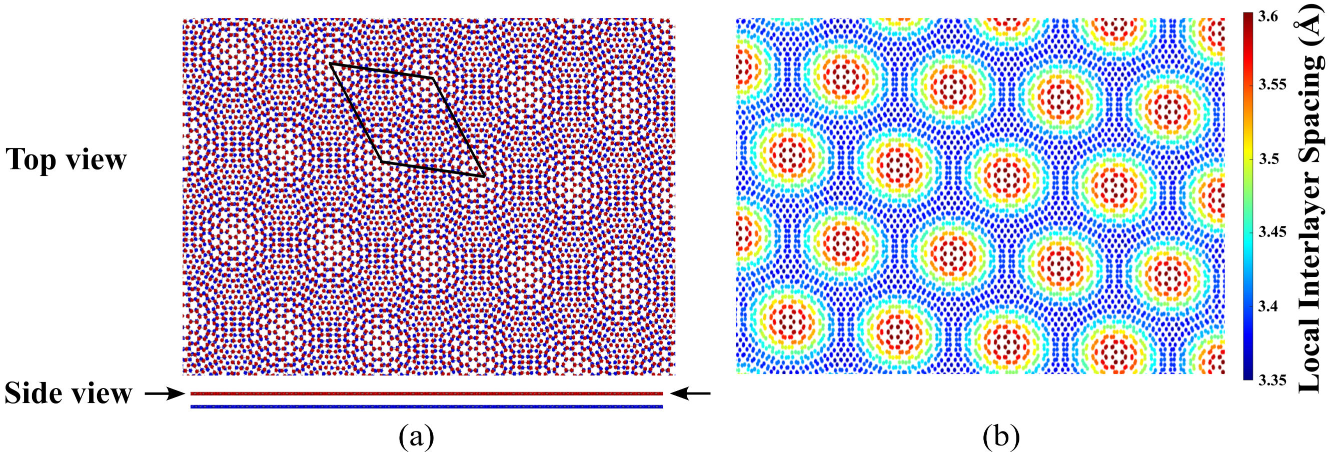

FIG. 1: (a) Relaxed atomistic structure and (b) interlayer spacing contour plot of θ= 6° TBG system under 1% uniaxial compressive strain.

TABLE I: Average ΔE_{ILE} threshold value considering five representative TBG systems (θ = 1.1°, 3.48°, 4.41°, 6° and 7.34°) in the presence of strain.

Strain (%)	ΔE_{ILE} (meV/atom)
0	8.24
+0.5	8.223
-0.5	8.21
+1	8.23
-1	8.207

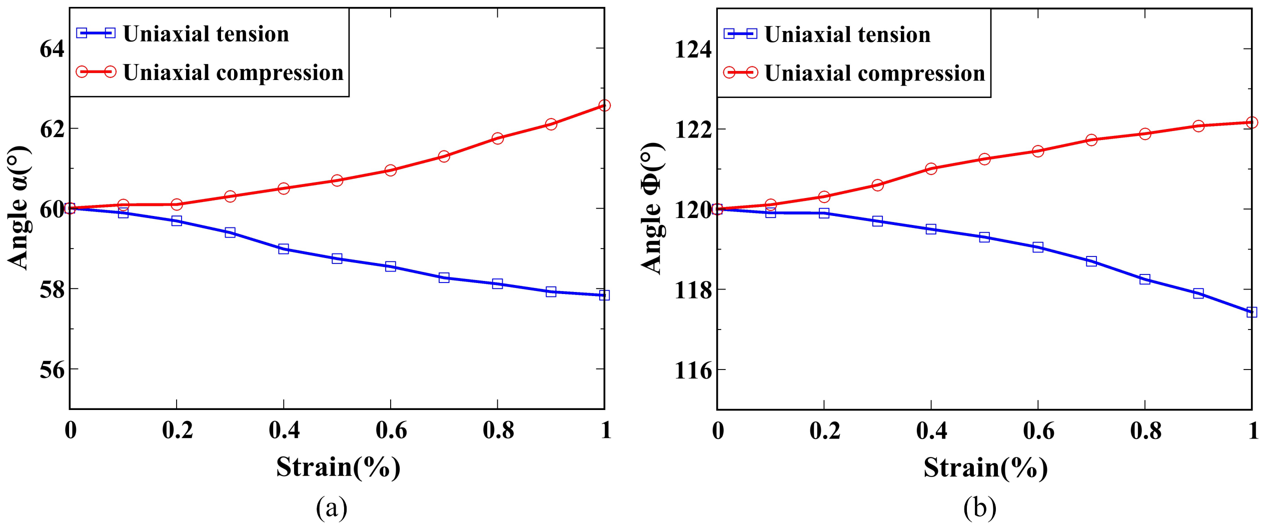

FIG. 2: Variation of angles α and ϕ with strain demonstrating the deformation of moiré patterns (for TBG system θ= 6°)

TABLE II: Evolution of area fractions f of local stacking domains with uniaxial tension and compression applied to the top layer

	θ = 1.1°			θ = 6°			θ = 13.2°		
Strain (%)	f_{AA}	f_{AB}	f_{SP}	f_{AA}	f_{AB}	f_{SP}	f_{AA}	f_{AB}	f_{SP}
0	0.135	0.474	0.391	0.25	0.39	0.36	0.272	0.379	0.349
+0.2	-	-	-	0.261	0.376	0.363	0.293	0.338	0.369
-0.2	-	-	-	0.239	0.407	0.354	0.257	0.399	0.344
+0.5	-	-	-	0.274	0.356	0.37	0.309	0.317	0.374
-0.5	-	-	-	0.218	0.432	0.35	0.239	0.419	0.342
+0.7	-	-	-	0.289	0.339	0.372	0.322	0.301	0.377
-0.7	-	-	-	0.2	0.455	0.345	0.218	0.443	0.339
+1	-	-	-	0.302	0.319	0.379	0.330	0.291	0.379
-1	-	-	-	0.188	0.471	0.341	0.2	0.462	0.338

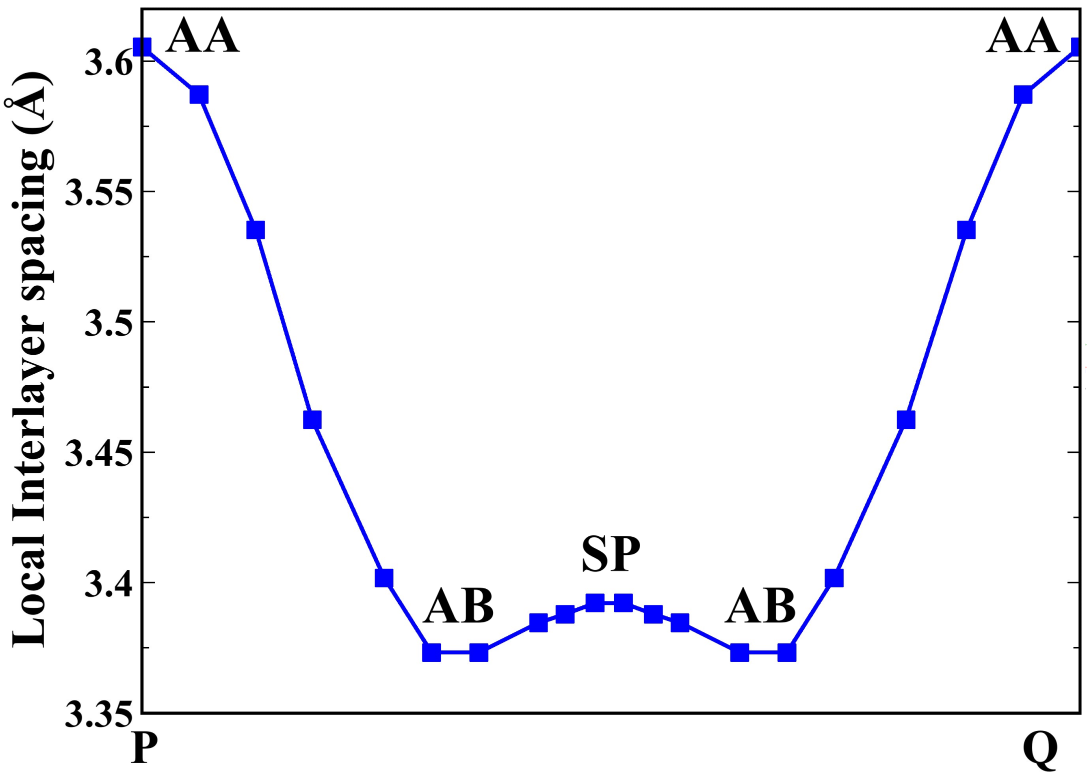

FIG. 3: Normalized spatial interlayer spacing difference (Δd) profiles traversing between centers of moiré pattern, i.e., path PQ in Fig. 2(a) (for TBG system θ= 6°)

TABLE III: Parameters for calculating β_{BOLS} pre-factors for TBGs and their respective sub-domains.

	$\theta = 1.1^\circ$				$\theta = 6^\circ$				$\theta = 13.2^\circ$			
Parameters	TBG	AA	AB	SP	TBG	AA	AB	SP	TBG	AA	AB	SP
d_z (Å)	1.406	1.40	1.405	1.411	1.424	1.417	1.423	1.43	1.438	1.431	1.437	1.441
z	5.008	4.88	4.987	5.12	5.43	5.185	5.409	5.612	5.851	5.67	5.792	5.911
C_z	0.913	0.909	0.912	0.916	0.926	0.918	0.924	0.929	0.933	0.929	0.933	0.935
E_z (eV)	0.775	0.783	0.776	0.768	0.752	0.764	0.751	0.741	0.73	0.743	0.733	0.727

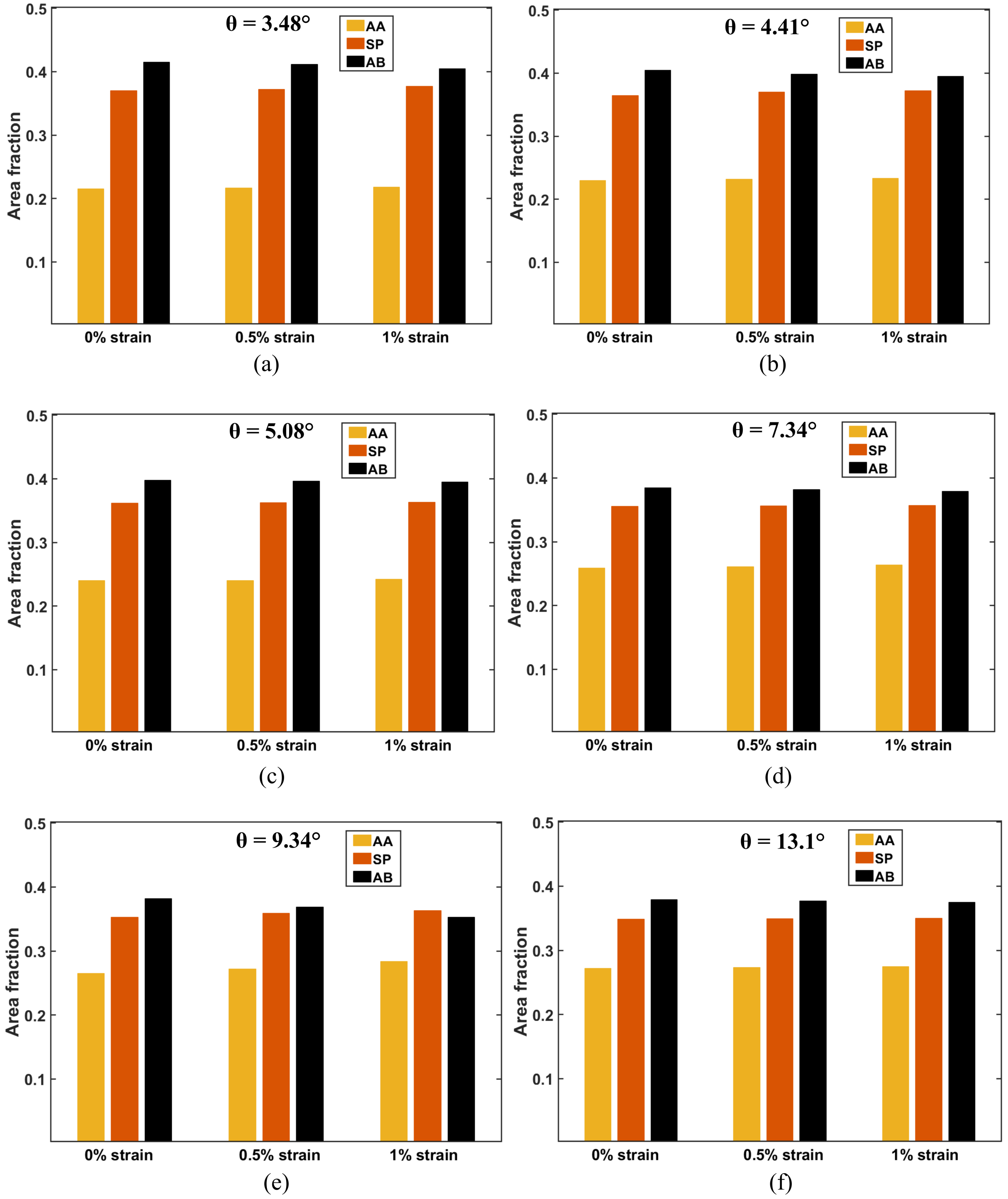

FIG. 4: Variation of area fractions of individual stacking domain with respect to heterostrain (tension) for different twist angles

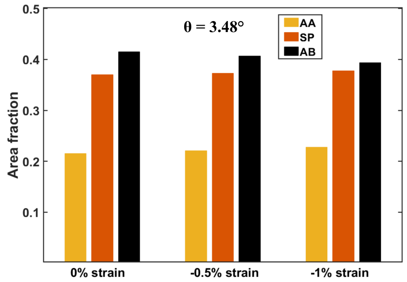

(a)

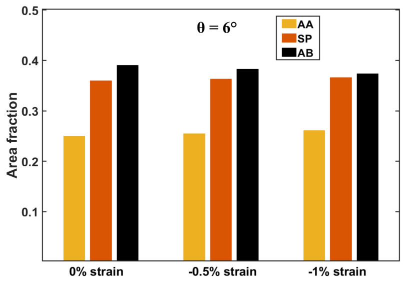

(b)

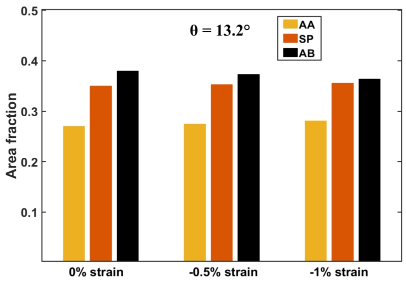

(c)

FIG. 5: Variation of area fractions of individual stacking domain with respect to heterostrain (compression) for $\theta = 3.48°$, 6° and 13.2°

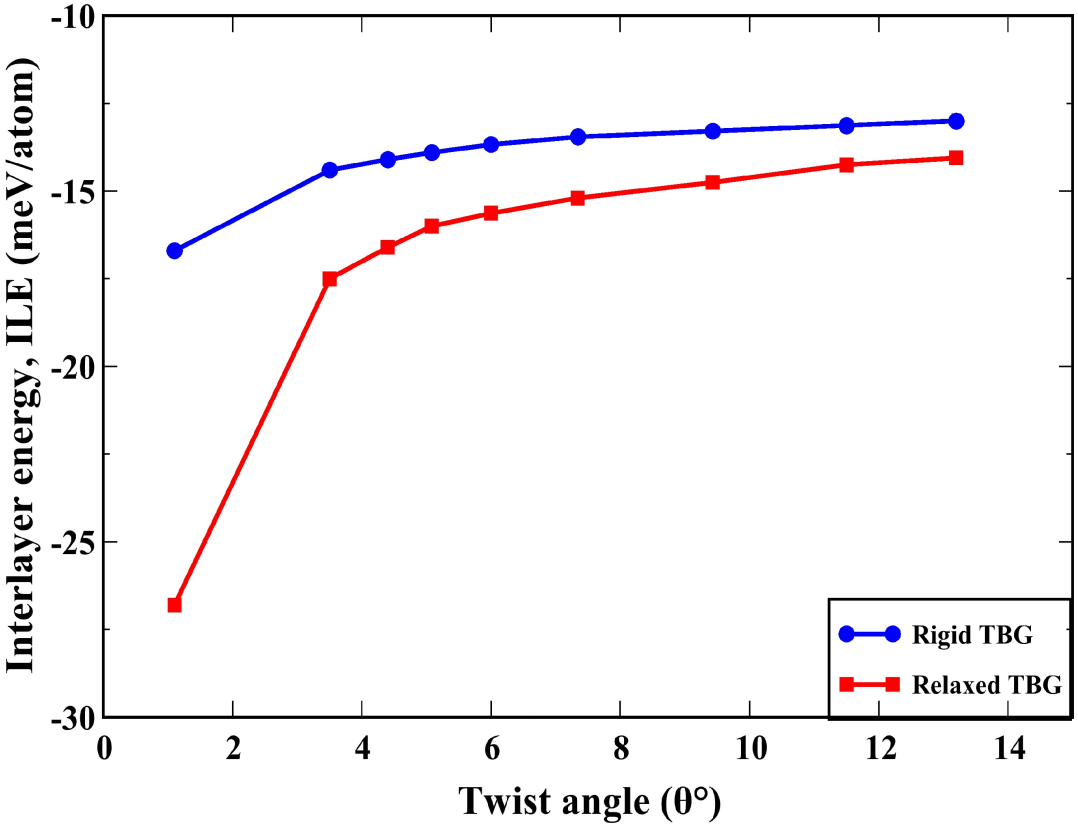

FIG. 6: Interlayer energy or vdW stacking energy for rigid and relaxed TBG systems. The ILE of relaxed TBG system is always lower than rigid TBG even for larger twist angles.

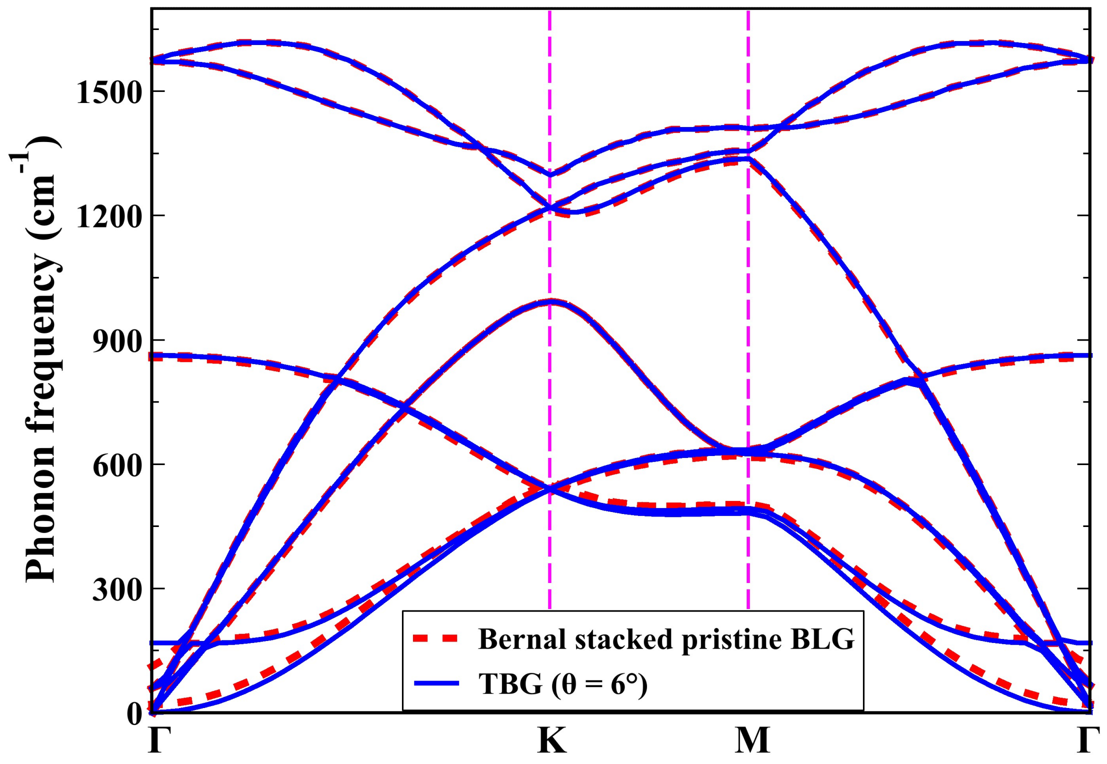

FIG. 7: Unfolded phonon spectra of TBG system $\theta = 6^{\circ}$ along high symmetry points of its Brillouin zone. Phonon dispersion spectra of Bernal stacked BLG is also shown for comparison.

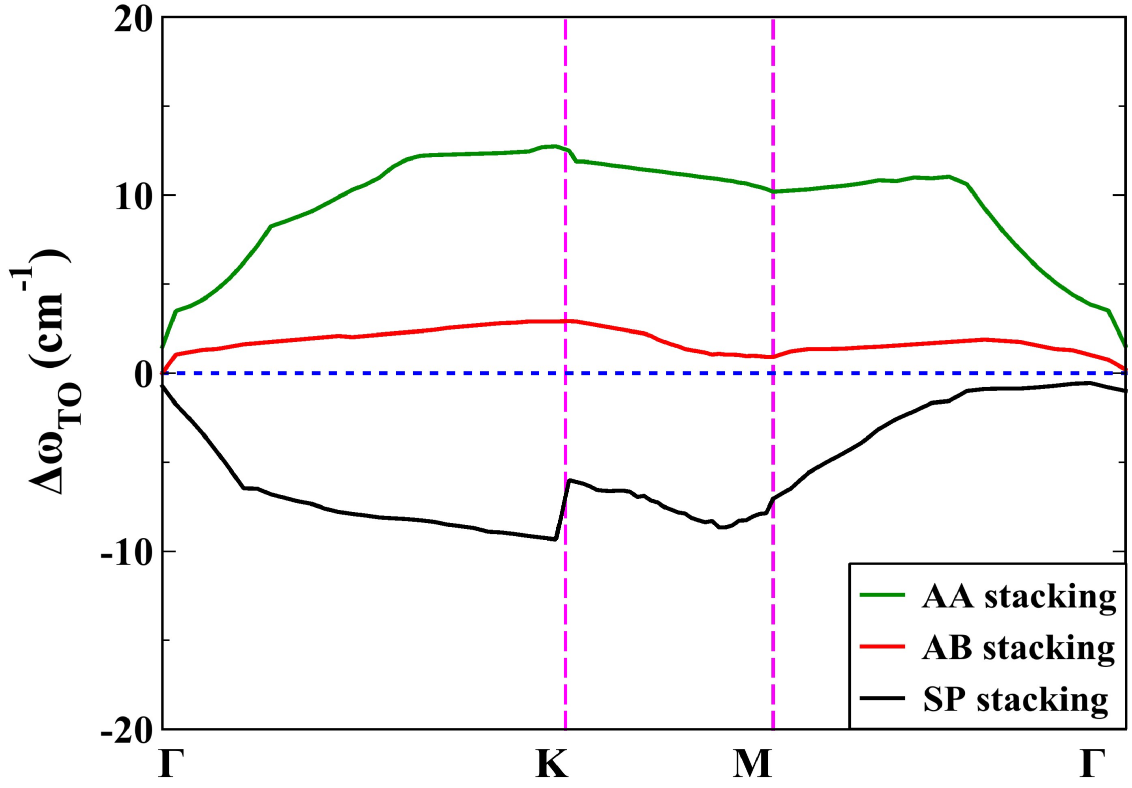

FIG. 8: Transverse optical (TO) phonon frequency difference with respect to TBG system $\theta = 6^{\circ}$

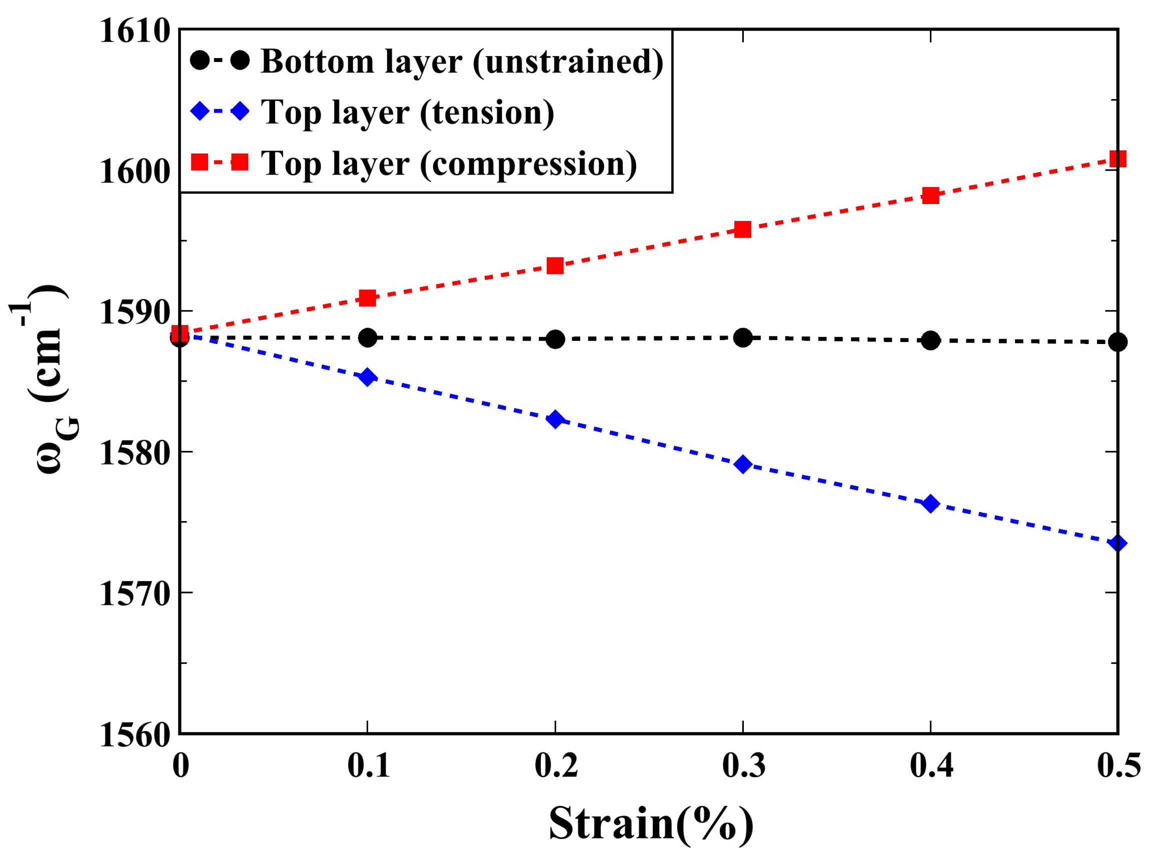

FIG. 9: BOLS predicted Raman G band frequencies of $\theta = 1.1^{\circ}$ TBG system as a function of applied heterostrain (tension and compression)

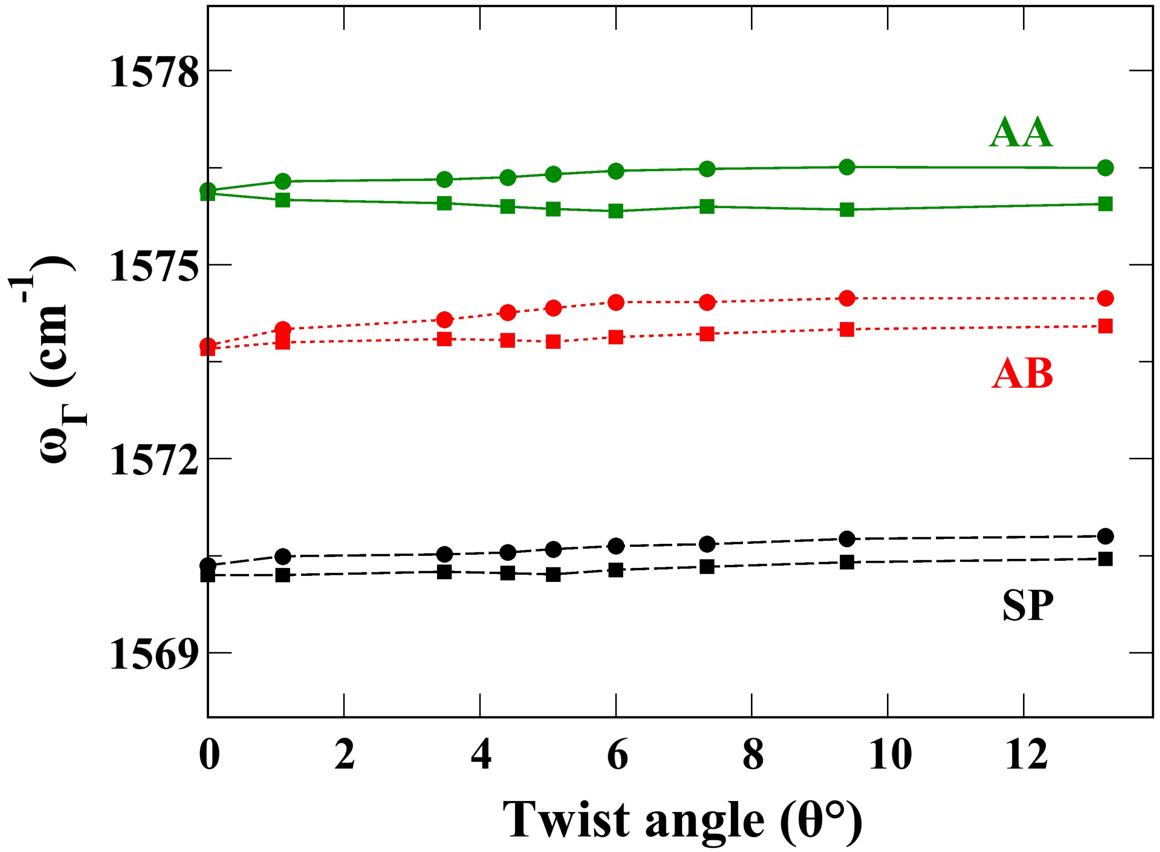

FIG. 10: High frequency LO and TO modes of individual sub-domains (AA, AB and SP) at Γ point as a function of twist angle.

TABLE IV: Calculated β^{ϵ}_{TBG} pre-factor values of strained top layer using BOLS parameters with respect to strain

Strain (%)	$\theta = 1.1^{\circ}$	$\theta = 6^{\circ}$	$\theta = 13.2^{\circ}$
0	3.135	3.306	3.466
+0.2	3.207	3.355	3.527
-0.2	3.078	3.214	3.421
+0.5	3.311	3.451	3.619
-0.5	2.988	3.064	3.356
+0.7	3.398	3.506	3.674
-0.7	2.732	2.961	3.312
+1	3.475	3.592	3.773
-1	2.602	2.795	3.248

REFERENCES

[1]A. Dey, R. Sharma, S. A. Dar, and H. H. Raza, "A computational investigation on structural, mechanical, electronic, magnetic, thermoelectric, and optical properties of crxpb (x= sc, ti) half-heusler alloys," Journal of Superconductivity and Novel Magnetism **34**, 781–796 (2021).

[2]A. Dey, B. A. Baraiya, S. Adhikary, and P. K. Jha, "First-principles calculations of the effects of edge functionalization and size on the band gap of be3n2 nanoribbons: Implications for nanoelectronic devices," ACS Applied Nano Materials **4**, 493–502 (2020).

[3]R. Sharma, A. Dey, S. A. Dar, and V. Srivastava, "A dft investigation of csmgx3 (x= cl, br) halide perovskites: Electronic, thermoelectric and optical properties," Computational and Theoretical Chemistry **1204**, 113415 (2021).

[4]É. D. Murray, K. Lee, and D. C. Langreth, "Investigation of exchange energy density functional accuracy for interacting molecules," Journal of Chemical Theory and Computation **5**, 2754–2762 (2009).

[5]A. Dey, R. Sharma, S. A. Dar, and I. H. Wani, "Cubic pbgeo3 perovskite oxide: A compound with striking electronic, thermoelectric and optical properties, explored using dft studies," Computational Condensed Matter **26**, e00532 (2021).

[6]A. Dey and D. Chakraborty, "Engineering the band structures of zigzag blue phosphorene and arsenene nanoribbons by incorporating edge corrugations: A first principles exploration," Journal of Nanoscience and Nanotechnology **21**, 5929–5936 (2021).

[7]V. Kumar, A. Dey, S. Thomas, M. A. Zaeem, and D. R. Roy, "Hydrogen-induced tunable electronic and optical properties of a two-dimensional penta-pt 2 n 4 monolayer," Physical Chemistry Chemical Physics **23**, 10409–10417 (2021).

[8]J. A. Abraham, R. Sharma, S. Ahmad, and A. Dey, "Dft investigation on the electronic, optical and thermoelectric properties of novel half-heusler compounds scaux (x= si, ge, sn, pb) for energy harvesting technologies," The European Physical Journal Plus **136**, 1091 (2021).

[9]D. W. Brenner, O. A. Shenderova, J. A. Harrison, S. J. Stuart, B. Ni, and S. B. Sinnott, "A second-generation reactive empirical bond order (rebo) potential energy expression for hydrocarbons," Journal of Physics: Condensed Matter **14**, 783 (2002).

[10]A. N. Kolmogorov and V. H. Crespi, "Registry-dependent interlayer potential for graphitic

systems," Physical Review B **71**, 235415 (2005).

[11]K. Zhang and E. B. Tadmor, "Energy and moiré patterns in 2d bilayers in translation and rotation: A study using an efficient discrete–continuum interlayer potential," Extreme Mechanics Letters **14**, 16–22 (2017).

[12]S. A. Chowdhury, K. Inzani, T. Peña, A. Dey, S. M. Wu, S. M. Griffin, and H. Askari, "Mechanical properties and strain transfer behavior of molybdenum ditelluride (mote2) thin films," Journal of Engineering Materials and Technology **144** (2022).

[13]X. Gao, H. Sun, D.-H. Kang, C. Wang, Q. J. Wang, and D. Nam, "Heterostrain-enabled dynamically tunable moiré superlattice in twisted bilayer graphene," Scientific reports **11**, 1–8 (2021).

[14]C. Androulidakis, E. N. Koukaras, G. Paterakis, G. Trakakis, and C. Galiotis, "Tunable macroscale structural superlubricity in two-layer graphene via strain engineering," Nature communications **11**, 1–11 (2020).

[15]A. Stukowski, "Visualization and analysis of atomistic simulation data with ovito–the open visualization tool," Model. Simul. Mater. Sci. Eng **18**, 015012 (2009).

[16]W. Zheng and C. Sun, "Energy environ. sci. 4, 627 (2011),".

[17]X. Yang, Y. Wang, J. Li, W. Liao, Y. Liu, and C. Q. Sun, "Graphene phonon softening and splitting by directional straining," Applied Physics Letters **107**, 203105 (2015).

[18]J. Campos-Delgado, L. G. Cançado, C. A. Achete, A. Jorio, and J.-P. Raskin, "Raman scattering study of the phonon dispersion in twisted bilayer graphene," Nano Research **6**, 269–274 (2013).

[19]Y. Hou, S. Zhang, Q. Li, L. Liu, X. Wu, and Z. Zhang, "Evaluation local strain of twisted bilayer graphene via moiré pattern," Optics and Lasers in Engineering **152**, 106946 (2022).

[20]V. Carozo, C. M. Almeida, E. H. Ferreira, L. G. Cancado, C. A. Achete, and A. Jorio, "Raman signature of graphene superlattices," Nano letters **11**, 4527–4534 (2011).

[21]F. Gargiulo and O. V. Yazyev, "Structural and electronic transformation in low-angle twisted bilayer graphene," 2D Materials **5**, 015019 (2017).

[22]A. I. Cocemasov, D. L. Nika, and A. A. Balandin, "Phonons in twisted bilayer graphene," Physical Review B **88**, 035428 (2013).

[23]F. Zheng and P. Zhang, "Phonon unfolding: A program for unfolding phonon dispersions of materials," Computer Physics Communications **210**, 139–144 (2017).

[24]D. Yoon, Y.-W. Son, and H. Cheong, "Strain-dependent splitting of the double-resonance

raman scattering band in graphene," Physical review letters **106**, 155502 (2011).

[25]A. Dey, R. Sharma, and S. A. Dar, "An extensive investigation of structural, electronic, thermoelectric and optical properties of bi-based half-huesler alloys by first principles calculations," Materials Today Communications **25**, 101647 (2020).

[26]A. Jorio and L. G. Cançado, "Raman spectroscopy of twisted bilayer graphene," Solid State Communications **175-176**, 3–12 (2013), special Issue: Graphene V: Recent Advances in Studies of Graphene and Graphene analogues.
